# Quantitative analysis of three-dimensional fibrillar collagen microstructure within the normal, aged and glaucomatous human optic nerve head

**DOI:** 10.1098/rsif.2015.0066

**Published:** 2015-05-06

**Authors:** H. J. Jones, M. J. Girard, N. White, M. P. Fautsch, J. E. Morgan, C. R. Ethier, J. Albon

**Affiliations:** 1 Optic Nerve Group, Cardiff Centre for Vision Science, Cardiff University, Cardiff, UK; 2 Bioimaging Labs, Cardiff University, Cardiff, UK; 3 Cardiff Institute for Tissue Engineering and Repair, Cardiff University, Cardiff, UK; 4 *In vivo* Biomechanics Laboratory, Department of Biomedical Engineering, National University of Singapore, Singapore; 5 Singapore Eye Research Institute, Singapore National Eye Centre, Singapore; 6 Department of Ophthalmology, Mayo Clinic, Rochester, NY, USA; 7 Georgia Institute of Technology and Emory, University School of Medicine, Atlanta, GA, USA; 8 Department of Bioengineering, Imperial College, London, UK

**Keywords:** optic nerve head, glaucoma, connective tissue, fibre orientation and alignment, small angle light scattering, second harmonic generation

## Abstract

The aim of this study was to quantify connective tissue fibre orientation and alignment in young, old and glaucomatous human optic nerve heads (ONH) to understand ONH microstructure and predisposition to glaucomatous optic neuropathy. Transverse (seven healthy, three glaucomatous) and longitudinal (14 healthy) human ONH cryosections were imaged by both second harmonic generation microscopy and small angle light scattering (SALS) in order to quantify preferred fibre orientation (PFO) and degree of fibre alignment (DOFA). DOFA was highest within the peripapillary sclera (ppsclera), with relatively low values in the lamina cribrosa (LC). Elderly ppsclera DOFA was higher than that in young ppsclera (*p* < 0.00007), and generally higher than in glaucoma ppsclera. In all LCs, a majority of fibres had preferential orientation horizontally across the nasal–temporal axis. In all glaucomatous LCs, PFO was significantly different from controls in a minimum of seven out of 12 LC regions (*p* < 0.05). Additionally, higher fibre alignment was observed in the glaucomatous inferior–temporal LC (*p* < 0.017). The differences between young and elderly ONH fibre alignment within regions suggest that age-related microstructural changes occur within the structure. The additional differences in fibre alignment observed within the glaucomatous LC may reflect an inherent susceptibility to glaucomatous optic neuropathy, or may be a consequence of ONH remodelling and/or collapse.

## 1. Introduction

Glaucoma is a multifactorial disease in which vascular and biomechanical mechanisms, in various combinations, have been proposed as important causes of retinal ganglion cell (RGC) axon death [1–5]. Recent studies have revealed the dynamic nature of glaucomatous optic neuropathy in which cells within the optic nerve head (ONH) are activated to respond to changes in their biomechanical and nutritional environment [4–9]. While age and intraocular pressure (IOP) remain the major risk factors, the observation that a high IOP is not always required for the initiation of glaucomatous damage raises the possibility that some aspects of ONH structure may predispose some eyes to RGC axon damage.

The lamina cribrosa (LC) is an important structural element in the ONH of the human eye and is most likely the site of RGC axonal damage in glaucoma [10], the second most common cause of bilateral irreversible blindness worldwide [11]. The LC comprises cribriform plates that offer vital structural support to approximately one million axons as they leave the eye. In glaucoma, the collapse, compression and rearrangement of the LC connective tissue beams is accompanied by RGC axon death and vision loss [1,12].

Sigal *et al.* [13] identified five influential determinants of ONH biomechanics, including the compliance of the sclera and LC. However, these models did not incorporate collagen microstructure, which is likely a key factor in peripapillary sclera (ppsclera) and LC biomechanics [14–17]; thus, a quantitative analysis of ONH collagen microstructure is warranted, and may be useful for e.g. identification of ONHs at risk of glaucoma damage. Previous studies have examined the two-dimensional organization of the LC and have biochemically analysed specific constituents of connective tissue and cellular components of the LC in human, monkey and rat ONHs [9,18–28]. However, only recently has research focused on the three-dimensional connective tissue architecture of the human ONH [29] and how this architecture affects biomechanical behaviour [30]. Although others have quantified the collagen fibre microstructure in the sclera and LC [17,31–33], none have analysed these simultaneously as a function of position in the ONH, of age and of glaucoma.

Here we analyse the three-dimensional fibre organization, regional distribution and degree of connective tissue fibre alignment in the human ONH to characterize the microarchitecture of the normal human LC and how these factors change as a function of age and glaucoma.

## 2. Material and methods

### 2.1. Source and preparation of optic nerve head tissue sections

Human ONHs were dissected from 21 globes with no history of ocular pathology (Corneal Transplant Service Bristol Eye Bank, UK) and three glaucomatous globes (Mayo Clinic, Rochester, USA; table 1), retaining information as to their orientation within the eye. All human tissue was enucleated and immersion fixed in 4% paraformaldehyde (PFA) within 48 h of donor death; then stored and used for research purposes.

**Table 1.** Demographics of donors of optic nerve head (ONH) samples used in ageing and glaucoma studies.

normal eyes						
transverse sections			longitudinal sections			
age (years)	sex	left/right eye	age (years)	sex	left/right eye	orientation^a^
young eyes						
2	M	left	11	F	right left	N-T S-I
6	M	left	21	M	right left	N-T S-I
21	F	left	57	M	right left	N-T S-I
elderly eyes						
88	F	left	62	M	right left	N-T S-I
88 (C1)^b^	F	right	78	M	right left	N-T S-I
87 (C2)^b^	M	left	82	F	right left	S-I N-T
85 (C3)^b^	F	left	88	M	right left	N-T S-I
**glaucoma eyes**						
**transverse sections**						
**age (years)**	**sex**	**left/right eye**	**mean deviation**^c^ **(dB)**	**cup/disc ratio**	**glaucoma severity**	
G1 86	M	right	−1.54 in 2002	unknown	mild	
G2 73	M	left	unknown	unknown	unknown, diagnosis in 2007	
G3 87	F	left	−12.81 in 2008	0.9	advanced	

^a^Longitudinal sections across the nasal–temporal (N-T) or superior–inferior (S-I) optic nerve axis, used in analysis of LC regions as a function of ageing.

^b^Elderly samples also used as controls in glaucoma ONH comparisons.

^c^Visual field mean deviation (MD).

Each ONH was snap frozen in liquid nitrogen-cooled isopentane (Fisher, UK). Serial 100 µm transverse cryosections were cut from seven normal ONHs (aged 2, 6, 21, 85, 87 and 88 years) and three glaucomatous ONHs, from prelamina to postlaminar optic nerve, using a sledge microtome (Microm HM 440E, Thermo Fisher, UK). Retinal tissue was trimmed until the choroid was visible which then allowed for fine adjustments of the microtome stage to be made with the blade orientation as a point of reference to ensure that the required sectioning angles were achieved. Longitudinal (160 µm) cryosections were also cut from seven ONH pairs (aged 11–88 years), with sections cut through the inferior–superior axis of one ONH of each pair, and through the nasal–temporal axis in the contralateral ONH. All sections were mounted in 1 : 1 PBS–glycerol.

### 2.2. Second harmonic imaging

Second harmonic generation (SHG) microscopy was performed on each ONH section using a META laser scanning microscope (Zeiss Ltd, UK), equipped with a 20× Plan-apochromat objective lens and multiphoton Ti:sapphire laser (Chameleon, Coherent UK Ltd, UK). Following excitation at 800 nm, the forward scattered signals from each ONH section were acquired as sequences of optical sections (256 × 256 × 1 pixel) at 1 µm increments of focus using an automated motorized stage. These were tiled together using LSM 510 v. 4.2 SP1 (Carl Zeiss, UK) to create three-dimensional image stacks of each ONH section. In this study, the latter was solely used to visualize the collagenous architecture of the ONH throughout each 100 µm tissue section so as to determine which sections contained prelamina, LC and/or postlaminar tissue. These three-dimensional image stacks were then converted to maximum intensity projections (MIPs) using ImageJ (1.45) software to ensure confident differentiation of each ONH region of interest, so that data selection and analysis for each region could be performed subsequently.

### 2.3. Small angle light scattering

Connective tissue fibre orientation and alignment were characterized using small angle light scattering (SALS) as previously described and validated by Girard *et al.* [31] In brief, the same tissue sections subjected to SHG were raster scanned, at 100 µm *x–y* intervals, using a 5 mW non-polarized HeNe laser (model 1125, JDS Uniphase, Milpitas, CA, USA, *λ*: 632.8 nm, beam diameter: 300 µm) and customized spatial-filter beam-shrinker assembly (modified from KT310/M; Thorlabs, Newton, NJ, USA). Light scattered from fibrous proteins within the samples was projected onto a diffuser screen behind the specimen, and collected using a CCD camera (1024 × 768 pixel, 8–16 bit, model B953; Pixelink, Ottawa, Ontario, Canada) with a red bandpass filter (model FL632.8–3; Thorlabs) positioned behind the diffuser screen.

### 2.4. Preferred fibre orientation and degree of fibre alignment fibre plots

**Figure 1.**
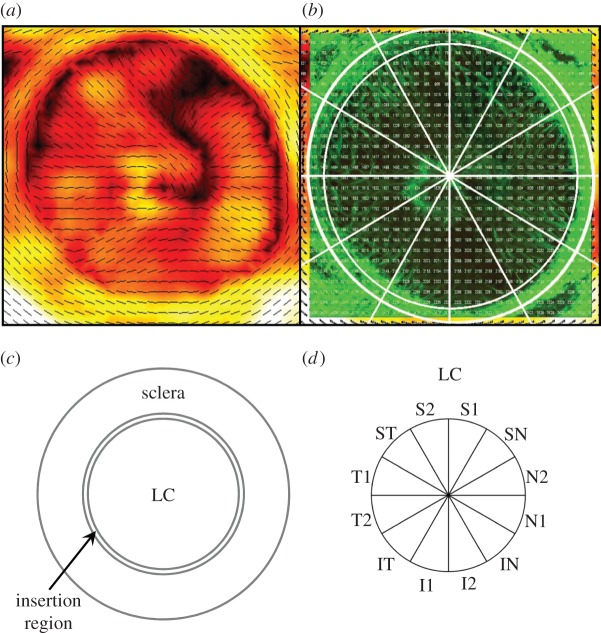
(*a*) A typical PFO/DOFA map of a human ONH. (*b*) A typical SHG image of the same ONH. PFO/DOFA maps were overlaid and aligned with SHG images to allow identification of the scleral canal margin in PFO/DOFA maps. (*c*) The ONH was subdivided into: the LC, terminating at the edge of the scleral canal; an insertion region, defined as an annular ring extending 150 µm from the scleral canal margin; and a peripapillary scleral region, defined as an annular ring extending from 150 to 1000 µm from the scleral canal margin. (*d*) The LC was subdivided into 12 regions for analysis. S, superior; N, nasal; I, inferior; T, temporal.

Connective tissue fibre distribution was determined from each scatter pattern using a custom written Matlab script (Mathworks, Natick, MA, USA), based upon a simplified Fraunhofer diffraction equation [31,34] to compute the preferred fibre orientation (PFO) and the fibre spread (OI), respectively, as determined [35] and described previously [31]. The degree of fibre alignment (DOFA) index, a value between 0 and 1, was then calculated as: 1—(OI/90°), where OI represented the fibre spread (measured in degrees). The DOFA index increases with the degree of fibre anisotropy from 0 (isotropy or random fibre alignment within the considered plane) to 1 (transverse isotropy or complete fibre alignment within the considered plane) [31,36]. The PFO and DOFA values of each SALS dataset from each ONH section were summarized as fibre distribution and colour gradient DOFA plots (figure 1*a*).

In order to determine the spatial distribution of DOFA and PFO values within different ONH regions, PFO and DOFA maps were aligned to SHG MIP images and grids depicting regions of interest and the individual SALS data point numbers (figure 1*b*) using Adobe PhotoShop v. 6.0.1 (Adobe, San Jose, CA, USA). Aligning the SHG MIPs to the fibre maps in this way allowed for accurate isolation of SALS data points within each ONH region of interest, which could not be accomplished using the PFO and DOFA maps only. Prior to image alignment, the scale of each PFO and DOFA map was calibrated (as determined by the number of pixels between the centres of each PFO line) so that it corresponded to the scale of its SHG image. If there was any ambiguity as to which region of interest an SALS data point was to be assigned, the data point was omitted from analysis. PFO and DOFA values within 12 regions of the LC relating to the superior, nasal, inferior and temporal regions (figure 1*d*) and DOFA values in the LC, region of LC insertion into the sclera (defined as a 150 µm thick annular ring adjacent to the scleral canal) and ppsclera (defined as an annular ring with inner and outer edges 150 and 1000 µm from the margin of the scleral canal, respectively; figure 1*c*) were analysed and compared.

### 2.5. Statistical analysis

Data were tested for normality using the Shapiro–Wilk test. Multivariate statistical analysis of DOFAs regionally and between different aged ONHs was carried out using a one-way ANOVA (with a Tukey post hoc test to account for type I errors) or Kruskal–Wallis depending on whether the data were normally distributed (SPSS v. PASW 18, SPSS Inc., USA). If the Kruskal–Wallis test was found to be significant (*p* < 0.05), multiple Mann–Whitney *U*-tests were performed to determine between which samples or regions the significant differences occurred. During these instances, the significance level was adjusted using the Bonferroni multiple testing correction method by dividing 0.05 by the number of Mann–Whitney *U*-tests.

To compare regional DOFA between each glaucomatous ONH and three age-matched controls, independent samples *t*-test or Mann–Whitney *U*-test pairwise comparisons were performed depending on whether the data were normally distributed. Again, the significance level was adjusted by the number of pairwise tests using the Bonferroni multiple testing correction method.

Pearson's correlation analysis was performed to determine the relationships between age and regional LC DOFAs within longitudinal ONH sections.

PFO regional data in normal and glaucoma LCs indicated a significant directional preference (*p* < 0.05 in Rayleigh's Uniformity test, Oriana circular statistics software, Kovach Computing Services, UK). The majority of data did not conform to von Mises distribution (*p* < 0.05, Watson's *U*^2^ one-sample test). Differences between the glaucomatous and each control LC PFO distribution, in each LC region, were assessed using the Mardia–Watson–Wheeler test with a Bonferroni adjusted significance level.

## 3. Results

### 3.1. Connective tissue fibre organization and alignment within the human optic nerve heads

**Figure 2.**
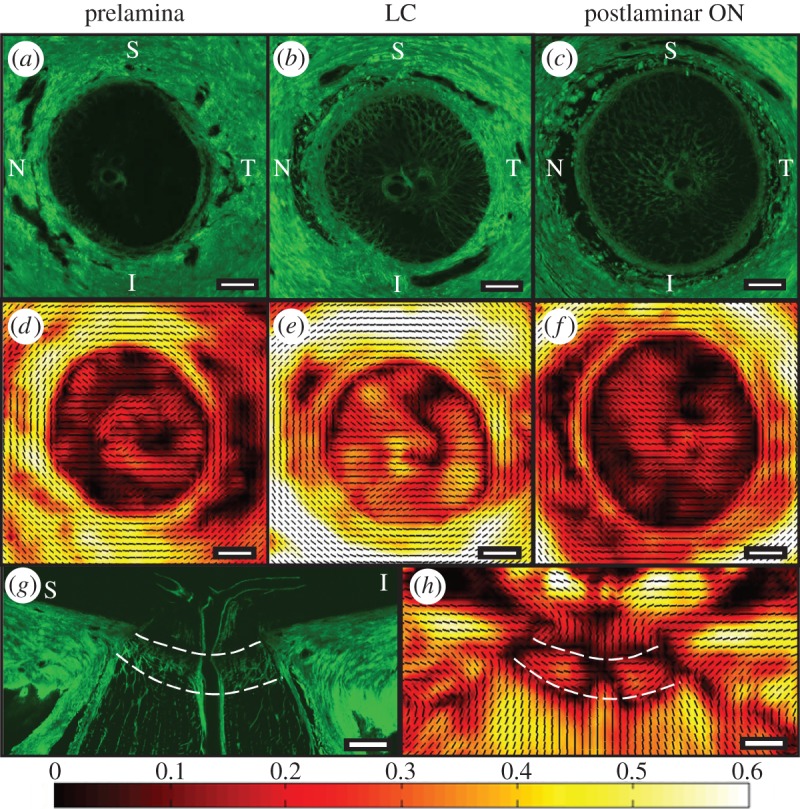
(*a*) SHG images from transverse ONH sections acquired from the prelamina, (*b*) LC and (*c*) postlaminar optic nerve (ON) from the left eye of an 88-year-old female donor. Corresponding PFO (black PFO orientation lines at 100 µm intervals) and DOFA (colour coded from black (0: poorly aligned) to white (0.6: highest alignment)) maps of the same (*d*) prelamina, (*e*) LC and (*f*) postlaminar optic nerve. (*g*) SHG image of a longitudinal ONH section (superior–inferior orientation) from the left eye of a 78-year-old male donor and (*h*) its corresponding PFO and DOFA map. The anterior and posterior LC boundaries are denoted by white dashed lines. Note: PFO is not meaningful when DOFA is pure black. S, superior; N, nasal; I, inferior; T, temporal. Scale bars, 500 µm.

Connective tissue fibre organization showed marked differences with anterior–posterior location within the ONH as indicated in representative images of different ONH regions within figure 2. In all regions, the DOFA is clearly higher outside of the scleral canal (figure 2*d–f*), where fibres have a preferred circumferential orientation around the scleral canal (figure 2*a*–*f*). This is explained by the circumferential arrangement of collagen fibres surrounding the scleral canal in the sclera (figure 2*a,b*,*d*,*e*) and, in the postlaminar region, within the dura mater (figure 2*c*,*f*).

The low level of alignment values indicated within the representative image of the prelamina (figure 2*d*) transverse tissue section reflects the fact that few fibres are present, as evidenced in the corresponding SHG image (figure 2*a*). This results in a very low level of light scatter that cannot be used to determine fibre alignment and orientation within this region.

A low level of alignment is also seen within the transversely cut postlaminar optic nerve (figure 2*f*). However, by contrast, a higher fibre alignment and a PFO of fibres running along the optic nerve axis are demonstrated in the longitudinal ONH sections as indicated in figure 2*g*,*h*.

The transverse orientations of fibres within the *en face* LC sections are shown in figure 2*b,e*. The fibre maps of the LC region within longitudinal ONH sections (figure 2*h*) indicate that these fibres are horizontally aligned across the ONH axis.

Figure 2*e* demonstrates the large variation of DOFAs that can be found within a single section of the LC. Nonetheless, the relatively low average magnitudes of the DOFA within the LC are consistent with the criss-cross appearance of LC beams and interweaving of fibrillar collagen, demonstrated in the SHG image (figure 2*b*). We note that, despite the low average DOFA in the LC, there are small LC regions in which the DOFA is higher than that within the prelamina (figure 2*d*) and postlaminar ON (figure 2*f*).

### 3.2. Effect of ageing on degree of fibre alignment within optic nerve head sections at the level of the lamina cribrosa

**Figure 3.**
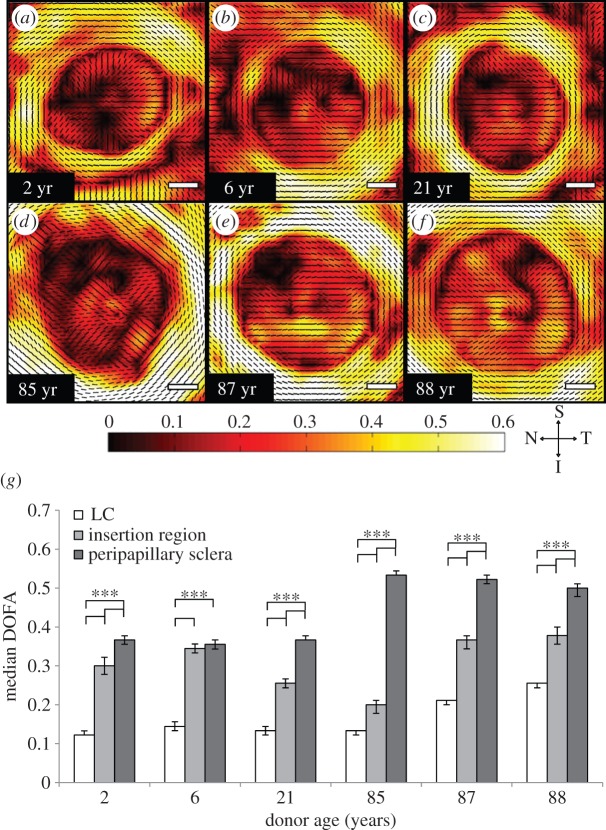
(*a*–*f*) DOFA colour maps of different aged ONHs indicate that there is an order of increasing fibre alignment from LC to insertion region to ppsclera. Scale bars, 500 µm. (*g*) Median (±95% CIs) DOFA was greatest in the ppsclera, compared with that within the region of insertion and LC (***p < 0.0003). The region of insertion DOFA was greater than that in the LC (***p < 0.0003). Bonferroni adjusted significant differences were determined using the Mann–Whitney *U*-test.

The trend of ppsclera DOFA > LC DOFA was apparent in transverse ONH sections at all ages (figure 3*a*–*f*). With the exception of the 6-year-old ONH, the median DOFA was the greatest in the ppsclera, compared with that in the region of insertion and in the LC (figure 3*g*, *p* < 0.0003). In addition, in all ages the DOFA within the insertion region was greater than that in the LC (figure 3*g*, *p* < 0.0003).

The DOFA within the ppsclera (in sections at the level of the LC) was significantly higher in the elderly ONHs, compared with that in the young ONHs (figure 3*g*, *p* < 0.00007). Interestingly, DOFA in the LC of the 87- and 88-year-old ONH, but not that of the 85-year-old ONH, was significantly greater than that in the young ONHs (figure 3*g*, *p* < 0.00007).

### 3.3. Regional differences in degree of fibre alignment within the lamina cribrosa

**Figure 4.**
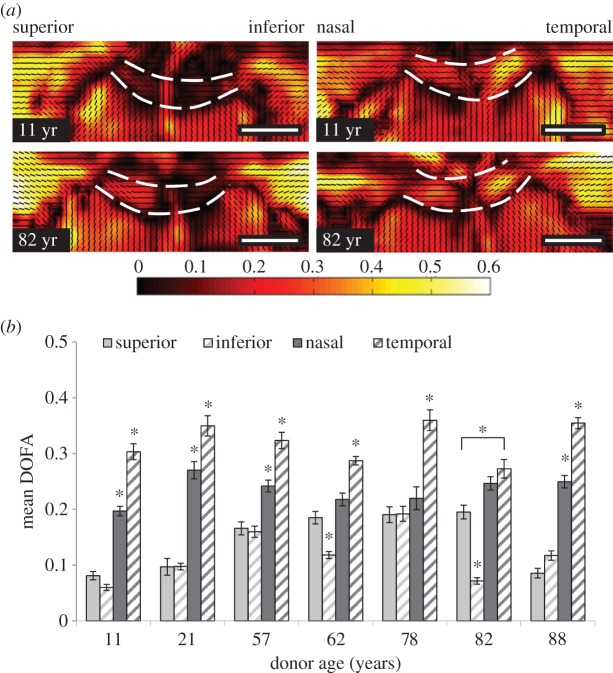
(*a*) SALS connective tissue DOFA (black: low alignment—white: higher alignment) and PFO (small black lines) fibre maps of longitudinal sections (superior–inferior and nasal–temporal orientation) of representative young (11 years) and elderly (82 years) human ONHs. Note the higher alignment (indicated by orange–yellow colour) in the temporal region in both aged ONHs. Anterior and posterior LC boundaries are denoted by the white dashed lines. Scale bars, 1000 µm. (*b*) Regional differences in DOFA (mean ± s.e.) within different aged (11, 21, 57, 62, 78, 82 and 88 years) LCs. DOFA was greatest in the temporal LC (*p < 0.001), and least in the superior and inferior regions within each ONH. Asterisk (*) denotes significant differences at *p* < 0.001 between a specific LC region and all other regions (unless indicated). Significant differences were determined using ANOVA with a Tukey post hoc test.

Regional differences in median DOFA were identified in longitudinal sections of human LC, (donor ages: 11, 21, 57, 62, 78, 82 and 88 years; table 1 and figure 4). The DOFA was greatest in the temporal region of all aged LC (figure 4*a*,*b*, *p* < 0.001), and higher in the nasal LC region, compared with superior and inferior regions. A trend towards an age-related increase in DOFA was observed up to 78 years (but with all data points included this was not significant, *p* = 0.188).

### 3.4. Degree of fibre alignment within glaucomatous optic nerve head sections at the level of the lamina cribrosa

**Figure 5.**
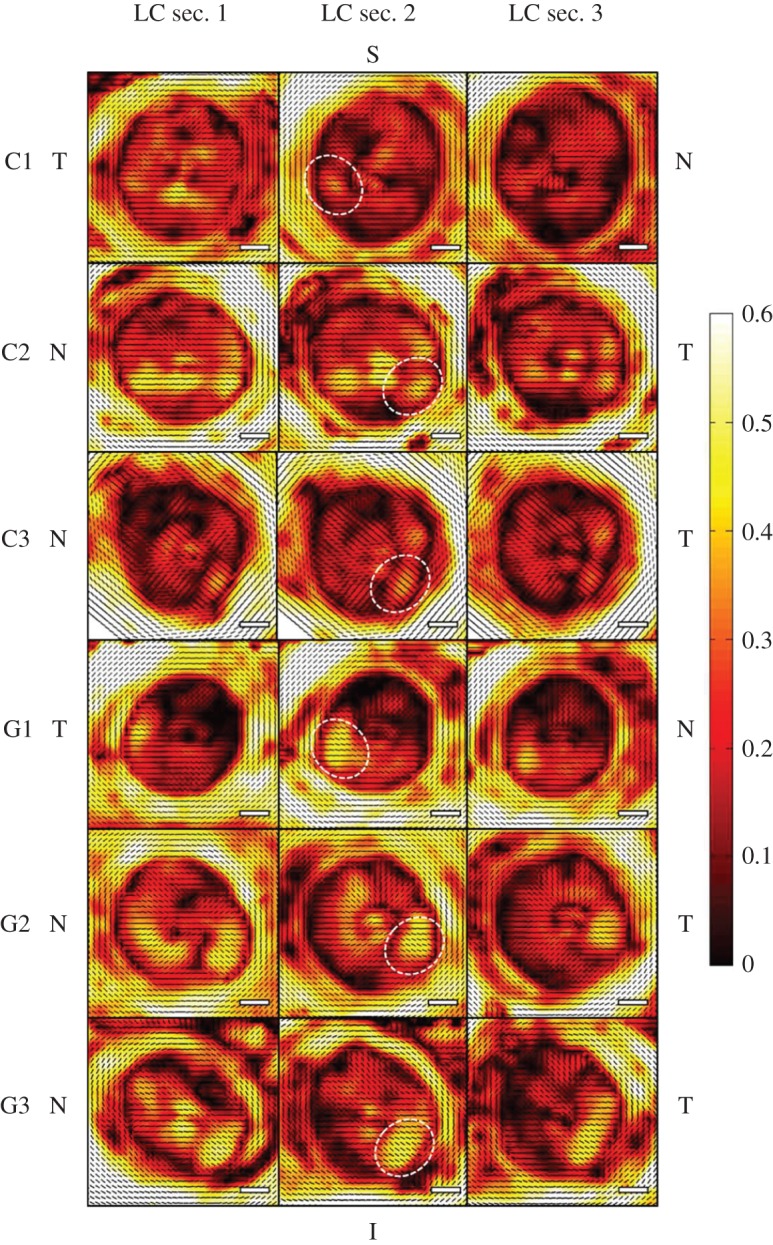
SALS DOFA (black, low alignment; white, high alignment) and PFO (small black lines) fibre maps derived from the transverse 100 µm ONH sections containing LC throughout their depth (anteriormost LC section 1 to LC section 3) of three glaucoma (G1, G2, G3) and three age-matched control ONHs (C1, C2, C3). In all ONHs, the order of DOFA was ppsclera > insertion region > LC. Small regions of higher fibre alignment were observed in the I-T LC quadrant (examples in each donor are denoted by white circles), which were more pronounced in the glaucoma LC sections. Orientation of ONHs is denoted by S, superior; N, nasal; I, inferior; T, temporal. Scale bars, 500 µm.

**Figure 6.**
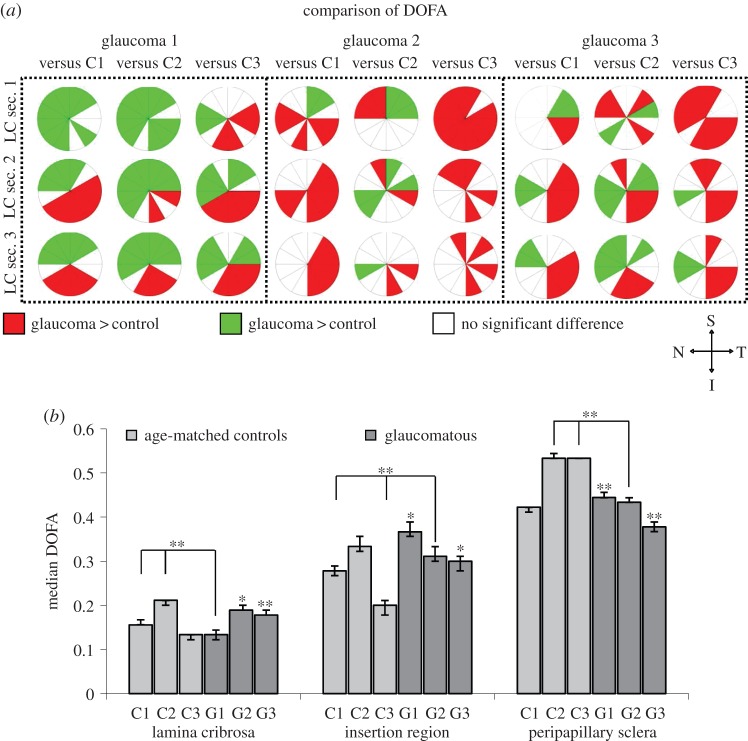
(*a*) Significant differences (*p* < 0.05) in DOFA between the three serial LC sections (anteriormost LC section 1 to LC section 3) from three glaucoma (G1, G2, G3) and three age-matched control (C1, C2, C3) LCs. Red: DOFA in glaucoma LC > control LC. Green: DOFA in glaucoma LC < control LC. White: no significant difference between glaucoma and control LC DOFA. Bonferroni adjusted significant differences were determined using the independent samples *t*-test or Mann–Whitney *U*-test. Note the orientation of LC sections, i.e. S, superior; N, nasal; I, inferior; T, temporal. (*b*) Comparison of median DOFAs (±95% CIs) in the LC, insertion region and ppsclera of glaucoma (G1, G2, G3, dark grey) and age-matched control (C1, C2, C3, light grey) ONHs. Unless the comparison is specified, significant differences (*p < 0.017, **p < 0.0033) in DOFA were found between the designated glaucoma LC and all control LCs. Bonferroni adjusted significant differences were determined using the Mann–Whitney *U*-test.

The distribution of DOFAs within individual transverse ONH sections of the three glaucomatous ONHs (figure 5, G1, G2 and G3) was similar to that observed in control ONHs (figure 5, C1, C2 and C3), with the order of DOFA as: ppsclera > insertion region > LC (figures 5 and 6*b*).

Interestingly, within each LC in transverse control and glaucomatous ONH sections (except section LC3 of C1), a small region of higher fibre alignment within the inferior–temporal (I-T) quadrant was observed (figure 5). These pockets of higher alignment appeared to be more pronounced in the glaucoma LC sections (G1, G2 and G3).

Although significant differences in DOFA between the glaucomatous and control LC sections were determined in many of the 12 regions compared (figure 6*a*), no consistent pattern could be discerned. However, the DOFA in I-T regions of glaucomatous LCs was significantly higher than the equivalent region in control LC sections (*p* < 0.017). This was true in the majority of G1 and G2 LC sections and in all G3 sections (figure 6*a*).

The ppsclera of all glaucoma samples were significantly less aligned than the ppsclera of C2 and C3 (figure 6*b*, *p* < 0.0033). However, when compared to C1 DOFA, the ppsclera was significantly more aligned in G1, less aligned in G3 and not significantly different in G2 (figure 6*b*). Additionally, ppsclera DOFAs were in the order of G1 > G2 > G3.

Within the insertion region, glaucoma samples G1 and G2 demonstrated a significantly higher DOFA than the controls (figure 6*b*, *p* < 0.017), with exception of C2 which was not significantly different from G2. The G3 insertion region was significantly more aligned than C1 and C3 but significantly less aligned then C2 (figure 6*b*).

### 3.5. Preferred connective tissue fibre orientation in young, old and glaucoma lamina cribrosa

Visual observation indicated that connective tissue fibres were oriented circumferentially in the ppsclera, with a horizontal orientation of fibres in the LC (figures 2*e*, 3*a*–*f* and 5). In longitudinal ONH sections, PFOs confirmed an orientation of connective tissue fibres across the scleral canal at the level of the LC (figures 2*h* and 4*a*). The beginning of the postlaminar optic nerve was clearly demarcated by a change in PFO, where fibres were oriented along the optic nerve axis (figures 2*h* and 4*a*).

**Figure 7.**
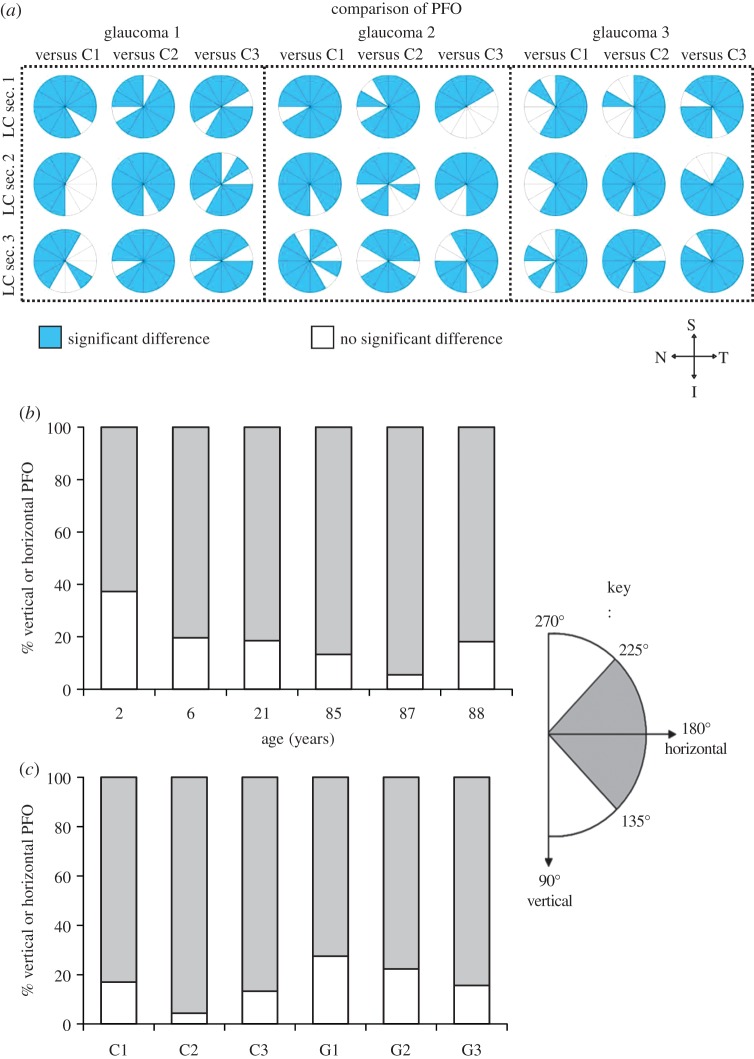
(*a*) Significant differences in PFO distributions (determined using the Mardia–Watson–Wheeler test; *p* < 0.017), denoted in blue, were identified in many of the 12 LC regions analysed in each of the three LC sections (anteriormost LC sec. 1 to LC sec. 3) between three glaucoma (G1, G2, G3) and three age-matched control (C1, C2, C3) LCs. Orientation of LC sections is shown; S, superior; N, nasal; I, inferior; T, temporal. (*b*) Percentage occurrence of temporal–nasal, i.e. horizontal (angles 135°–225°; grey bars), and superior–inferior, i.e. vertical (angles 90°–135° and 225°–270°; white bars), PFOs in an age range of LCs and (*c*) in PFOs in transverse sections through the LC of control (C1, C2, C3) and glaucoma (G1, G2, G3) ONHs. In all LCs, a horizontal fibre orientation predominated. Angle range is provided in key.

Significant differences were observed between the PFO distributions in the glaucomatous and control eyes in most of the 12 LC regions analysed throughout the LC depth (figure 7*a*, *p* < 0.05). PFOs within the LC of transverse ONH sections were assigned as those with a superior–inferior orientation, termed ‘vertical’ (i.e. 90°–135° and 225°–270°), or temporal–nasal orientation, termed ‘horizontal’ (i.e. 135°–225°). In transverse ONHs, analysis of the PFOs within the LC of young (2, 6 and 21 years), elderly (85, 87 and 88) and glaucoma ONHs indicated that connective tissue fibres were preferentially oriented with a horizontal distribution across the LC (i.e. nasal to temporal, figure 7*b*,*c*). Specifically, in all ONHs, normal and glaucoma, 63–96% of connective tissue fibres had a predominantly horizontal orientation.

## 4. Discussion

While previous studies have quantified ONH collagen fibre microstructure [15,17,31,32,37], this study is the first to quantify it as a function of age and glaucoma, simultaneously in both the LC and ppsclera of human eyes. These data are central to our understanding of ONH biomechanical behaviour, as collagen fibre microstructure is generally a key factor influencing connective tissue biomechanics.

Within the ONH, fibre alignment was highest in the ppsclera, compared to the region of insertion and LC. The ring of circumferential collagen fibrils in the ppsclera is consistent with previous studies of human ONHs [29,33] and has been proposed as a mechanism to limit IOP-related expansion of the scleral canal and in-plane tensile strains, as demonstrated by computational modelling [14–16,38].

The insertion region had a DOFA that was intermediate between that of the adjacent ppsclera and LC. The LC is considered a weak point in an otherwise continuous cornea–scleral shell. The insertion region may provide a region of adjustment between the ‘weak’ LC and ‘tough’ sclera and therefore support the LC in resisting IOP-related stress [39] without damage to LC connective fibres or their disinsertion from the tougher, denser sclera. However, as yet it is unclear whether a lower alignment within the insertion region is protective or a weakness in design. Thus finite-element modelling is currently being undertaken to provide biomechanically relevant information regarding this interesting pattern.

The DOFA within the ppsclera (at the level of the LC) increased with age. In contrast, a previous SALS study indicated no age-related changes in fibre alignment within the sclera [37]. The contrast in findings between the current study and the study by Yan *et al.* [37] is likely due to the difference in sampling of sclera position and depth. In the latter study, Yan *et al.* evaluated fibre alignment in a 1 cm^2^ tissue patch through the full sclera depth from the temporal side of the scleral canal. Our current study analysed an 850 µm annulus of the scleral canal within three individual 100 µm thick tissue sections, at the level of the LC. Fibre anisotropy has previously been shown to vary through the scleral depth with peak anisotropy occurring at depths between 200 and 500 µm [33]. Therefore, age-related changes in ppsclera fibre alignment may also be depth dependent and occur in response to age-related changes in the adjacent LC. The higher DOFAs in the elderly ONHs are likely related to changing ONH composition [20–22], and mechanical properties [40] with age. Like the LC, the sclera also stiffens with age [41–45] and has high non-enzymatic cross-linking [46]. Although age is a major risk factor in glaucoma, the role of a stiffer ONH and any increased susceptibility to glaucomatous damage has yet to be determined, and is currently the focus of research in glaucoma therapy [47].

Within the adult LC, fibrillar collagen and elastin represent approximately 45% and 28%, of tissue dry weight, respectively, localized to the cribriform plates and at the region of insertion into the ppsclera [21,22]. Transverse SHG images demonstrated the interweaving network of LC collagen fibrillar bundles in ONH transverse sections, radiating outwards from the connective tissue of the central retinal vessels. In transverse views of the LC, this translated into a low DOFA due to the criss-cross arrangement of fibres. In all transverse LC views (young, old and glaucomatous), most fibres were horizontally oriented along the nasal–temporal axis, consistent with previous *in vivo* identification of a horizontal central ridge [48]. This was consistent with observations made in longitudinal sections, where fibre alignment was higher than that in transverse LC sections. This preferred alignment, as in other tissues, e.g. tendon, will likely assist the LC in resisting tensional forces exerted in the direction of fibre alignment and limit scleral canal expansion. In addition, it may contribute to the proposed regional susceptibility of RGC axons to glaucomatous damage [18,19]. The change in fibre anisotropy from LC to postlaminar optic nerve septa within the longitudinal sections could also act as a demarcation of the posterior LC boundary which is currently difficult to define. This observation may be useful in future LC thickness studies following improvement of current optical coherence tomography (OCT) approaches [49].

When glaucomatous ONH sections were compared to control ONHs, differences in DOFA and PFO were observed, but these did not follow a consistent pattern. A previous wide angle X-ray scattering study by Pijanka *et al.* [33] found that the superior–temporal and inferior–nasal quadrants of the ppsclera of normal eyes were less aligned than the corresponding quadrants in glaucomatous eyes. A study by Danford *et al.* [32] also found regional differences in fibre organization between the normal and glaucomatous sclera, although this study investigated an area that was approximately 2.25 mm from the edge of the scleral canal. The lack of consistent differences between the glaucomatous and control ppsclera within the current study may therefore be due to the fact that, in contrast to the studies mentioned, the data from the annulus of the ppsclera was analysed.

The glaucomatous LC, with a visual field mean deviation of −1.54 dB was significantly less aligned than the glaucomatous LC with a mean deviation of −12.84 dB, consistent with an increase in anisotropy in the LC of advanced glaucoma eyes. The ‘advanced’ glaucomatous ONH demonstrated significantly less fibre alignment in the insertion region and ppsclera compared to the ‘mild’ glaucomatous ONH. This could be a result of previous changes in the glaucomatous ONH (e.g. progressive changes include LC plate compression, deformation and disorganization) [3,18,50,51] and LC remodelling (new synthesis and/or degradation of connective tissue components) [7–12,52]. However, we cannot conclude that fibre alignment and orientation provides evidence of glaucoma progression in this study, as only three glaucoma ONHs were studied. Further studies, with an increased number of ONHs at different glaucoma stages are required to clarify the importance of fibre alignment in the glaucomatous LC. As we all know, this type of research material is very difficult to find; thus it is important to present these findings, so that these data can be compared and contrasted to other published data as it appears.

A key finding, of potential clinical importance, is the observation of foci of higher DOFA, in the I-T quadrant in most LCs, which were significantly more prominent in glaucomatous LCs. In glaucoma, focal defects predominate in this LC quadrant [53], and it is a common site for optic disc haemorrhages [54]. Additionally, glaucomatous neuroretinal rim loss is also more pronounced at the I-T disc margin [55–57]. Quigley & Addicks [18] have previously indicated this ONH region has greater susceptibility to nerve damage in early glaucoma, and Winkler *et al.* [29] have demonstrated that the I-T LC has a low collagen density compared with other LC regions. It remains to be seen if these I-T pockets of high alignment, which appear to advance in glaucoma, are indicators of glaucomatous ONH susceptibility and/or markers of disease progression or are a result of a compensation mechanism due to a disordered structure in other ONH regions. One mechanism may be an adaption of anisotropy within the I-T LC in response to the significant age-related softening of the I-T ppsclera previously indicated by Fazio *et al.* [45].

Further investigations will need to be carried out to confirm the exact reason for the observed changes in DOFA between young, old and glaucomatous ONHs. An increase in sample size is essential to statistically confirm that changes in DOFA are due to age/disease and not to inter-donor biological variation. Although the eyes in this study were not fixed under pressure prior to enucleation, which may slightly influence collagen microarchitecture (primarily crimp), all eyes were processed in the same way, allowing meaningful comparisons. Future microstructural analysis will determine the age- and glaucoma-related changes that underlie these differences in DOFA.

A limitation of this study is that two-dimensional projections from the ONH sections are being used to assess a three-dimensional tissue. Analyses of the microarchitecture are therefore subjected to inherent errors common to two-dimensional techniques such as differences in sectioning angle and do not account for the natural curvature of the LC. However, the use of both serial transverse and longitudinal ONH sections has provided meaningful results within these two structural planes that can be used as a basis for analysis in future three-dimensional ONH reconstructions.

In conclusion, the combination of SALS and SHG imaging is an effective tool for the quantification of fibre alignment and orientation throughout the *ex vivo* ONH. The SHG images are essential to confirm whether diffuse scatter from which alignment parameters were calculated are due to real fibre isotropy and to determine whether connective tissue fibres are actually present within the region being analysed. This study highlights the importance of the interpretation of SALS data in conjunction with an imaging technique that allows for the visualization of the tissue components.

Additionally, if the I-T region within the LC could be probed *in vivo* it may prove to be an invaluable biomarker for optic nerve susceptibility to glaucoma and determination of disease stage. Advances in high-resolution imaging techniques, such as OCT, raise the possibility that this region could be analysed *in vivo* for the detection of those discs at risk of glaucoma damage [58–60].

## Data accessibility

Owing to the large size of the SALS raw data (*ca* 10 GB per section), these may be requested from the corresponding author by emailing albonj@cardiff.ac.uk. Owing to the sensitive nature of human-derived data, we will only be able to release data in an anonymized form.
